# Prediction of COVID-19 Infections for Municipalities in the Netherlands: Algorithm Development and Interpretation

**DOI:** 10.2196/38450

**Published:** 2022-10-20

**Authors:** Tjeerd van der Ploeg, Robbert J J Gobbens

**Affiliations:** 1 Faculty of Health, Sports and Social Work Inholland University of Applied Sciences Amsterdam Netherlands; 2 Zonnehuisgroep Amstelland Amstelveen Netherlands; 3 Department Family Medicine and Population Health Faculty of Medicine and Health Sciences University of Antwerp Antwerp Belgium; 4 Tranzo Tilburg School of Social and Behavioral Sciences Tilburg University Tilburg Netherlands

**Keywords:** municipality properties, data merging, modeling technique, variable selection, prediction model, public health, COVID-19, surveillance, static data, Dutch public domain, pandemic, Wuhan, virus, public, infections, fever, cough, congestion, fatigue, symptoms, pneumonia, dyspnea, death

## Abstract

**Background:**

COVID-19 was first identified in December 2019 in the city of Wuhan, China. The virus quickly spread and was declared a pandemic on March 11, 2020. After infection, symptoms such as fever, a (dry) cough, nasal congestion, and fatigue can develop. In some cases, the virus causes severe complications such as pneumonia and dyspnea and could result in death. The virus also spread rapidly in the Netherlands, a small and densely populated country with an aging population. Health care in the Netherlands is of a high standard, but there were nevertheless problems with hospital capacity, such as the number of available beds and staff. There were also regions and municipalities that were hit harder than others. In the Netherlands, there are important data sources available for daily COVID-19 numbers and information about municipalities.

**Objective:**

We aimed to predict the cumulative number of confirmed COVID-19 infections per 10,000 inhabitants per municipality in the Netherlands, using a data set with the properties of 355 municipalities in the Netherlands and advanced modeling techniques.

**Methods:**

We collected relevant static data per municipality from data sources that were available in the Dutch public domain and merged these data with the dynamic daily number of infections from January 1, 2020, to May 9, 2021, resulting in a data set with 355 municipalities in the Netherlands and variables grouped into 20 topics. The modeling techniques random forest and multiple fractional polynomials were used to construct a prediction model for predicting the cumulative number of confirmed COVID-19 infections per 10,000 inhabitants per municipality in the Netherlands.

**Results:**

The final prediction model had an *R*^2^ of 0.63. Important properties for predicting the cumulative number of confirmed COVID-19 infections per 10,000 inhabitants in a municipality in the Netherlands were exposure to particulate matter with diameters <10 μm (PM10) in the air, the percentage of Labour party voters, and the number of children in a household.

**Conclusions:**

Data about municipality properties in relation to the cumulative number of confirmed infections in a municipality in the Netherlands can give insight into the most important properties of a municipality for predicting the cumulative number of confirmed COVID-19 infections per 10,000 inhabitants in a municipality. This insight can provide policy makers with tools to cope with COVID-19 and may also be of value in the event of a future pandemic, so that municipalities are better prepared.

## Introduction

COVID-19 was first identified in December 2019 in the city of Wuhan, China. The World Health Organization [1] declared the outbreak a public health emergency of international concern on January 30, 2020, and a pandemic on March 11, 2020. The virus quickly spread and is still among us. After infection, symptoms such as fever, a (dry) cough, nasal congestion, and fatigue can develop. COVID-19 can have many adverse outcomes such as a reduction in the quality of life of children, adolescents [2], and older adults, especially when they have to live in a lockdown [3]. In some cases, the virus causes severe complications such as pneumonia and dyspnea and can result in death. In a retrospective study among critically ill patients with COVID-19 admitted to intensive care units in Italy, both the mortality rate and absolute mortality were high [4].

**Figure 1:**
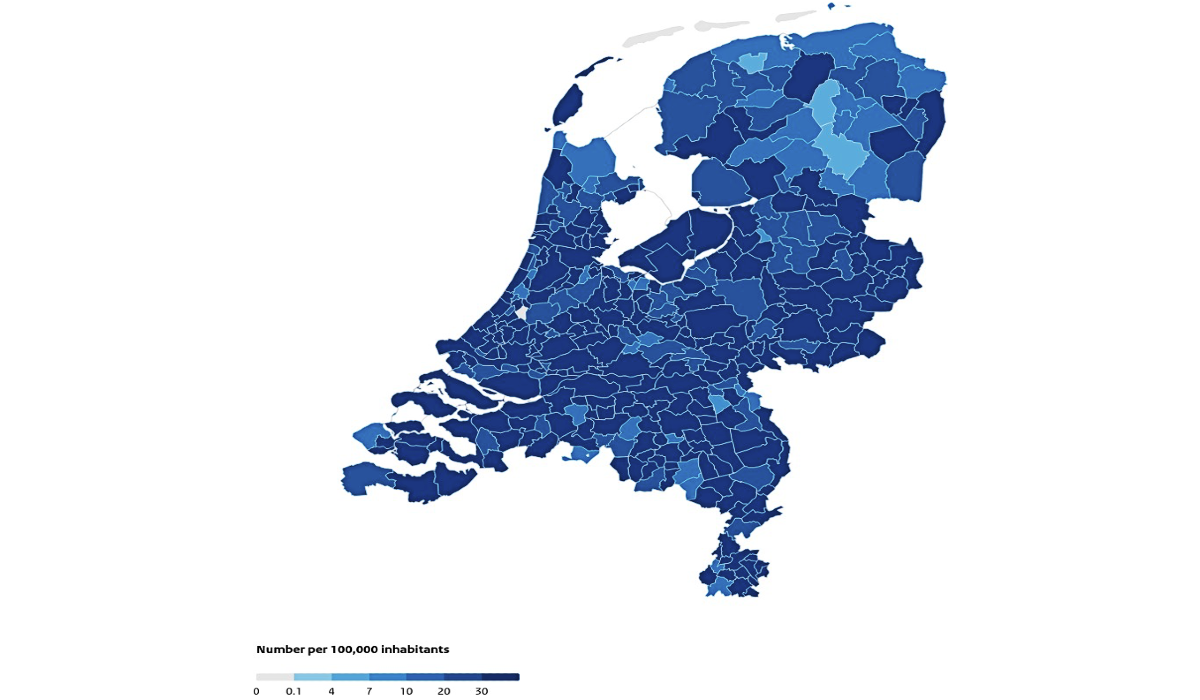
Cumulative confirmed infections per municipality (reproduced with permission from the National Institute for Public Health and the Environment [5]) .

At the end of February 2020, the first COVID-19 case in the Netherlands was confirmed. In June 2020, 46,000 cases had been identified. On May 9, 2021, 1,406,517 infections were confirmed [5]. However, the distribution of the confirmed infections over the Dutch municipalities was not uniform. There were differences between the municipalities with respect to the number of confirmed infections (see Figure 1). This finding raised the question of why some municipalities were hit so hard by COVID-19. A cross-sectional study conducted in the United States showed that states that have denser populations, lower socioeconomic status, and lower mean age are associated with higher incidence rates of COVID-19 [6].

Some studies identified the degree of air pollution, especially the presence of particulate matter with diameters <10 μm (PM10) and nitrogen dioxide (NO_2_), as an important factor for the high number of confirmed infections [7]. Currently, older adults face the most threats and challenges, not only in the Netherlands but also in many countries. Although all age groups are at risk of contracting COVID-19, older adults are more vulnerable to developing severe illness due to physiological changes that come with aging and potential underlying health conditions [1].

A Dutch study hypothesized that religious gatherings and the number of confirmed infections were associated [8]. This study showed that in the Dutch bible belt, church attendance was strongly related to the number of confirmed infections. However, in Southern Netherlands, a traditionally Catholic part of the Netherlands, nominal church membership mattered more than church attendance. Based on these findings, the study concluded that religious gatherings probably facilitated the spread of the virus in both a direct and indirect way.

Differences between urban and rural areas were found in a Brazilian study, demonstrating that in urban areas, more people were infected with COVID-19 [9]. However, in a sample of 5009 American adults, it was shown that rural residents were less likely to participate in COVID-19–related preventive behaviors, including working from home and wearing a face mask in public [10]. Thus, more infections in rural areas would be expected.

An American study identified political party affiliation as a factor associated with the spread of COVID-19 [11]. In this study of young adults, who lived predominantly in Los Angeles County or elsewhere in California, self-reported Republican political party affiliation was associated with less frequent physical distancing and participating in social recreational activities that may perpetuate the COVID-19 pandemic.

We aimed to predict the cumulative number of confirmed COVID-19 infections per 10,000 inhabitants in a municipality using the properties of 355 municipalities in the Netherlands. In the Netherlands, the National Institute for Public Health and the Environment [5] and the Central Agency for Statistics [12] are important data sources for daily COVID-19 numbers and information about municipalities, respectively. With these data, we can gain insight into the relationship between municipality properties and the cumulative number of confirmed infections per 10,000 inhabitants in a municipality. This insight can provide policy makers with tools to cope with COVID-19 and may also be of value in the event of a future pandemic, so that municipalities are better prepared. It is even conceivable that different protective measures may need to be taken in municipalities or regions.

## Methods

### Data Collection

Our aim was to predict the cumulative number of confirmed COVID-19 infections per 10,000 inhabitants in a municipality using the properties of 355 municipalities in the Netherlands. Therefore, we retrieved data from the National Institute for Public Health and the Environment and the Central Agency for Statistics and merged these data into a database consisting of 335 municipalities and variables with respect to the municipality topics as listed in Textbox 1, supplemented with the cumulative number of confirmed infections per 10,000 inhabitants in a municipality. All variables within the topics were expressed as numbers or fractions.

**Municipality topics.**

**Topics**

- Age distribution
- Dependency ratio
- Ethnicity
- Degree of urbanization
- Cause of death
- Household type
- Education level
- Social benefit
- Number of cars or motor bikes
- Number of facilities
- Health
- Number of caregivers
- Mean distances to facilities
- Political party preference
- Labor force participation
- Number affiliated to sports club
- Exposure to air pollution
- Illiteracy
- Benchmark scores for the municipalities
- Religion

### Outcome Variable and Modeling

The outcome variable in this study was the cumulative number of confirmed infections per 10,000 inhabitants in a municipality in the period from January 1, 2020, to May 9, 2021. We used a technique based on random forest (RF) for backward variable selection (VARSELRF) [13] to select important variables for the prediction of the outcome. Next, we used RF for the calculation of the importance of the selected variables [14]. The selected variables were then used for the construction of a multiple fractional polynomials (MFP) model for predicting the outcome. The MFP modeling technique offers the opportunity for building models with functions of the relevant explanatory variables and is more flexible than linear regression [15].

### Description of the Modeling Techniques

#### RF Modeling Technique

RF is an ensemble classifier that consists of many decision trees. In the case of classification, RF outputs the class that is the mode among the classes from individual trees. In the case of regression, RF outputs the value that is the mean of the values outputted from individual trees. Each tree is constructed using a bootstrap sample from the original data. A tree is grown by recursively partitioning the bootstrap sample based on the optimization of a split rule. In regression problems, the split rule is based on minimizing the mean squared error, whereas in classification problems, the Gini index is commonly used. At each split, a subset of candidate variables is tested for the split rule optimization, similar to recursive partition modeling [16]. For prediction, a new sample is pushed down the tree. This procedure is iterated over all trees in the ensemble. Key parameters are the number of trees and the number of candidate variables [14].

#### VARSELRF Modeling Technique

VARSELRF is a variable selection technique based on RF with backward stepwise elimination of variables that are not important [13,17]. This variable selection technique returns very small sets of predictor variables and will not return sets of variables that are highly correlated, because they are redundant.

#### MFP Modeling Technique

The MFP modeling technique is a collection of functions targeted at the use of fractional polynomials for modeling the influence of continuous predictor variables on the outcome in regression models, as introduced by Royston and Altman [18] and modified by Sauerbrei et al [15,19]. It combines backward elimination with a systematic search for a transformation to represent the influence of each continuous predictor variable on the outcome.

### Performance Measures

We used the *R*^2^ statistic, the root of the mean squared error (RMSE), and the normalized RMSE (NRMSE) as measures for the performance of the prediction model. The *R*^2^ measures how well the predictor variables can explain the variation in the outcome variable. The RMSE measures the typical distance between the predicted value from the prediction model and the value of the outcome variable. The NRMSE is the ratio of the RMSE of the prediction model and the RMSE of the model with no predictor variables (RMSE_0_).



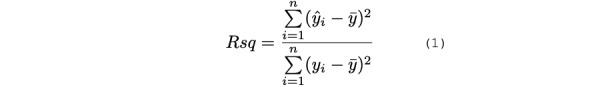





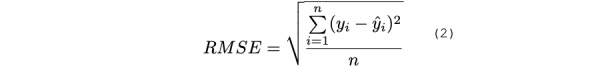





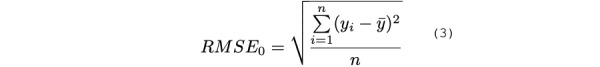





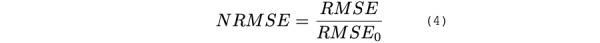



In the formulas, *y* represents the actual values, *yˆ* represents the prediction for the actual values from the prediction model, *y¯* represents the mean value of the actual values, and n represents the number of cases. An *R*^2^ value toward 1 indicates good performance, whereas an *R*^2^ value toward 0 indicates bad performance. An NRMSE value toward 0 indicates good performance [20].

### Data Analysis

For all analyses, we used R statistical software (version 3.4.4; R Foundation for Statistical Computing) [21].

### Ethical Consideration

For this study, no ethical approval was required because the granularity of the data was at the municipality level.

## Results

Table 1 shows percentiles with respect to the cumulative number of confirmed infections, hospital admissions, and deaths per 10,000 inhabitants. The median (50% percentile) of the cumulative number of confirmed infections per 10,000 inhabitants—our outcome variable—was 884. Of the 335 municipalities, 25% (n=84) had 1022 cumulative confirmed infections per 10,000 inhabitants or more.

**Table 1:** Cumulative numbers per 10,000 inhabitants (May 9, 2021).

Percentile	Confirmed infections, n	Hospital admissions, n	Deaths, n
2.5%	511	6	3
25%	757	11	7
50%	884	15	9
75%	1022	20	13
97.5%	1278	32	21

Table 2 shows the top 10 municipalities with respect to the cumulative number of confirmed infections, hospital admissions, and deaths per 10,000 inhabitants. It is noteworthy that the top 10 municipalities for confirmed infections did not overlap with the top 10 municipalities for hospital admissions and deaths. It is also noteworthy that the first 3 municipalities for confirmed infections are known as Christian municipalities and that the first 3 municipalities for hospital admissions are located in the southern part of the Netherlands.

Table 3 shows the selected variables of the VARSELRF modeling technique and the ranking by importance score with the RF modeling technique. The importance scores were calculated by the RF modeling technique; a higher score means that the variable is more important. The variables “Exposure to PM10” and “Labour party PvdA” had the highest importance scores compared to the other variables in Table 3.

Table 4 shows the coefficients of an MFP model with the selected variables. The *R*^2^ of the model was 0.63 as calculated by (1), and the NRMSE of the model was 0.61 as calculated by (4).

For example, suppose that 20% of people in a municipality voted for the Liberal party Democraten 66. Using the transformation of this predictor variable and the coefficients in Table 4, the contribution of this predictor variable to the prediction of the cumulative number of confirmed infections per 10,000 inhabitants is then calculated as:



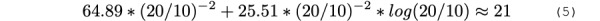



Similarly, the contributions of the other predictor variables can be calculated with the transformations of the predictor variables and their coefficients in Table 4. Table 5 shows the characteristics, example values, and the contributions of the predictor variables. By summing the contributions in Table 5 and the intercept in Table 4, the prediction of 837 cumulative confirmed infections per 10,000 inhabitants is obtained using the example values.

**Table 2:** Top 10 municipalities with cumulative numbers per 10,000 inhabitants (May 9, 2021).

Municipality		Cumulative number per 10,000 inhabitants, n
**Confirmed infections**		
	Bunschoten	1873
	Hardinxveld-Giessendam	1428
	Maasdriel	1348
	Edam-Volendam	1336
	Tubbergen	1310
	Bladel	1309
	Zaltbommel	1291
	Horst aan de Maas	1285
	Katwijk	1279
	Nederweert	1278
**Hospital admissions**		
	Boekel	38
	Peel en Maas	38
	Cranendonck	37
	Oudewater	37
	Bernheze	35
	Gouda	35
	Uden	33
	Gemert-Bakel	32
	Landerd	32
	Eijsden-Margraten	30
**Deaths**		
	Bernheze	25
	Zandvoort	25
	Cranendonck	24
	Krimpen aan den IJssel	23
	Laren	23
	Boxtel	22
	Gouda	22
	Heemstede	22
	Boekel	21
	Capelle aan den IJssel	21

**Table 3:** Selected variables (May 9, 2021).

Variables	Variable importance score
Exposure to PM10^a^	13,083
Labour party PvdA^b^	8544
Animal welfare party PvdD^c^	4164
Denomination or philosophical grouping	3578
Age-class 20-25 (years)	3445
Households with children	3361
Liberal party D66^d^	3166
Catholic	3034
Green party GroenLinks	3023

^a^PM10: particulate matter with diameters <10 μm.

^b^PvdA: Partij van de Arbeid.

^c^PvdD: Partij voor de Dieren.

^d^D66: Democraten 66.

**Table 4:** Coefficients of the multiple fractional polynomials model (May 9, 2021).

Variables with transformations	Coefficient
Intercept	−355.78
(Exposure to PM10^a^/10)^1^	669.95
(Households with children/100)^1^	696.71
(Liberal party D66^b^/10)^−2^	64.89
(Liberal party D66/10)^−2^ × log[(Liberal party D66 / 10)]	25.51
(Age-class 20-25/10)^−2^	−34.98
(Catholic/100)^1^	282.44
(Denomination or philosophical grouping/100)^1^	−344.94
(Animal welfare party PvdD^c^)^−2^	195.17
(Labour party PvdA^d^/10)^1^	−74.90
(Green party GroenLinks/10)^1^	−109.32

^a^PM10: particulate matter with diameters <10 μm.

^b^D66: Democraten 66.

^c^PvdD: Partij voor de Dieren.

^d^PvdA: Partij van de Arbeid.

**Table 5:** Characteristics predictor variables and example values.

Variables	Minimum	Mean	Maximum	Example values	Contribution
Exposure to PM10^a^	15.1	18.6	21.4	19.0	1272.9
Households with children	19.0	35.4	57.6	37.1	258.5
Liberal party D66^b^	0.5	10.8	23.2	20.0	20.6
Age-class 20-25 (years)	3.2	5.5	16.2	4.1	−208.1
Catholic	0.4	32.2	88.3	63.0	177.9
Denomination or philosophical grouping	19.9	57.5	98.1	68.6	−236.6
Animal welfare party PvdD^c^	0.2	2.7	6.0	2.4	35.0
Labour party PvdA^d^	0.2	5.2	10.6	4.8	−36.1
Green party GroenLinks	0.2	7.1	20.3	8.4	−91.6

^a^PM10: particulate matter with diameters <10 μm.

^b^D66: Democraten 66.

^c^PvdD: Partij voor de Dieren.

^d^PvdA: Partij van de Arbeid.

## Discussion

### Overview

The COVID-19 pandemic has endangered human lives all over the world. It has led to health care problems (physical, psychological, and social). The World Health Organization points out that measures such as self-isolation and quarantine may lead to an increase in loneliness, depression, anxiety, and self-harm or suicidal behavior [22]. In addition, COVID-19 puts a lot of pressure on the health care system and ensures an economic slowdown in all countries involved [23]. In this study, we aimed to study the properties of 355 municipalities in the Netherlands for predicting the cumulative number of confirmed COVID-19 infections per 10,000 inhabitants in a municipality.

Relevant static data per municipality were collected from data sources that were available in the Dutch public domain, and these data were merged with the dynamic daily number of infections in the period from January 1, 2020, to May 9, 2021 [5,12]. We used the VARSELRF [13] technique (based on RF) to select important variables, followed by MFP modeling to develop a prediction model for the cumulative number of confirmed infections per 10,000 inhabitants in a municipality.

### Principal Findings

Our prediction model explained 63% of the variance of the dependent variable (cumulative number of confirmed COVID-19 infections per 10,000 inhabitants). This finding means that our prediction model is useful for predicting the cumulative number of confirmed infections per 10,000 inhabitants in a municipality in the Netherlands. In our study, we used 20 municipality topics for developing a prediction model. The most important predictors were exposure to PM10, being a Labour party voter, and the number of children in households.

### Comparison With Prior Work

A systematic review identified 7 models for the identification of people at risk for COVID-19 in the general population. In these models, the most frequently included predictors were age, comorbidities, vital signs, and image features [24].

In our study, exposure to PM10 had the highest importance score out of all predictors. Other studies also observed significant associations of PM10 with COVID-19 infections [25,26]. Social distancing led to a 35.56% and 20.41% decrease, relative to the previous year, in the mean of PM10 and NO_2_, respectively [27]. However, a systematic review showed that exposure to particulate matter with diameters <2.5 μm and NO_2_ provided a more important contribution to triggering COVID-19 spread and lethality than PM10 [28]. All findings indicate reducing air pollution as a current public health problem, as well as in a more sustainable post–COVID-19 world [27].

Our study showed that being a Labour party (Partij van de Arbeid) voter can be considered an important predictor of the cumulative number of COVID-19 infections per 10,000 inhabitants. One explanation could be that voters for left-wing parties conformed more to government rules (eg, social distancing) than voters for right-wing parties (who have more distrust about government actions) [29]. Labour party voters are also generally older [30]. Support for the Labour party is stronger in the northern areas of the Netherlands. We recommend further studies focusing on the characteristics of these party voters, including lifestyle characteristics, to better understand the association with the cumulative number of COVID-19 infections per 10,000 inhabitants.

In this study, the number of children in households was an important predictor of the cumulative number of COVID-19 infections per 10,000 inhabitants. This finding may be due to school attendance [31]. However, a study among 300,000 adults showed that the risk of COVID-19 requiring hospital admission was reduced as the number of children in households increased [32]. Moreover, no association was found between exposure to children and COVID-19 [32]. This finding is confirmed by a systematic review, which concluded that it is unlikely that children are the main drivers of this pandemic [33]. Additionally, in this study, the top 10 municipalities for confirmed infections did not overlap with the top 10 municipalities for hospital admissions and deaths as we had expected. This finding indicates that there is no association between the cumulative number of COVID-19 infections per 10,000 inhabitants and both adverse outcomes, which stands in contrast to other studies [4,34,35]. From the top 10 municipalities for hospitalizations and deaths, 3 municipalities appeared on both lists. This result was expected, as only people with severe COVID-19 complaints are admitted to hospital, and these people will have an increased risk of death [36]. COVID-19 vaccines can be considered the most promising means of reducing the spread of this virus. Thus, it is crucial that many people get vaccinated. A scoping review including 22 studies showed that gender, age, education, and occupation were associated with vaccine acceptance [37]. In addition, trust in authorities, vaccine efficacy, vaccine safety, having a current or previous influenza vaccination, and perceived risk of a COVID-19 infection were also associated with COVID-19 acceptance [37]. It is important for all countries to address the barriers to vaccine acceptance, so that maximum vaccine coverage can be achieved. This step requires the commitment of all stakeholders, from policy makers to health care professionals and scientists.

### Limitations

Some limitations of our study should be mentioned. Our prediction model was only based on data of municipalities in the Netherlands, so the external validity of the model is limited. The model may not be usable elsewhere in the world. This limitation will certainly apply to countries outside of Europe that have a different culture, health care, and political systems and inhabitants with different sociodemographic and health characteristics. Unfortunately, other prediction models of COVID-19 have a similar problem [24,38]. For preventing and combatting COVID-19, it is vital to apply these prediction models on different data sets and check changes in the behavior of the models from one country to another. A second limitation refers to the presence of several COVID-19 variants [39]. The Omicron variant emerged in 2021, and the developed prediction model may not be applicable to this variant. A third limitation is that variables such as the presence of childcare for families impacted by COVID-19 or other public health factors were not available in our data set. This limitation will be taken into account for follow-up research.

### Conclusions

In conclusion, collecting data about municipality topics in relation to the cumulative number of confirmed infections in a municipality can give insight into the most important topics for predicting the number of cumulative confirmed infections per 10,000 inhabitants for a municipality in the Netherlands. In the prediction model, the most important topics were exposure to PM10, being a Labour party voter, and the number of children in households. The findings contribute to increasing our knowledge on COVID-19 and can provide policy makers with tools to cope with COVID-19. This study may also be of substantive value in the event of a future pandemic, so that municipalities are better prepared. It is even conceivable that different protective measures may need to be taken in municipalities or regions.

## Abbreviations

MFP: multiple fractional polynomials
NO_2_: nitrogen dioxide
NRMSE: normalized root of the mean squared error
PM10: particulate matter with diameters <10 μm
RF: random forest
RMSE: root of the mean squared error
VARSELRF: variable selection based on random forest

## Data Availability

The data set used and analyzed during the current study is available from the corresponding author on reasonable request.
